# *trans*-Diaqua­bis{1,3-bis­[5-(2-pyrid­yl)-2*H*-tetra­zol-2-yl]propane}zinc(II) bis­(perchlorate)

**DOI:** 10.1107/S1600536808006703

**Published:** 2008-03-14

**Authors:** Hugo Gallardo, Fernando Molin, Adailton J. Bortoluzzi, Ademir Neves

**Affiliations:** a Depto. de Química, Universidade Federal de Santa Catarina, 88040-900 Florianópolis, Santa Catarina, Brazil

## Abstract

The Zn^II^ ion in the title compound, [Zn(C_15_H_14_N_10_)(H_2_O)_2_](ClO_4_)_2_, lies on a centre of symmetry. The distorted N_4_O_2_ octa­hedral coordination environment around the Zn atom is composed of two 1,3-bis­[5-(2-pyrid­yl)-2*H*-tetra­zol-2-yl]propane ligands (*L*1) and two water mol­ecules, coordinated in *trans* positions. The ligand acts as a typical bidentate chelating ligand through one of its 2-pyridyl-2*H*-tetra­zole units, forming a five-membered Zn—N—C—C—N metallacycle with a small N—Zn—N bite angle [77.40 (8)°]. The other 2-pyridyl-2*H*-tetra­zole unit remains uncoordinated. The average Zn—N distance (2.156 Å) is somewhat longer than the distance between the Zn^II^ center and the aqua ligand [2.108 (2) Å]. The coordinated pyrid­yl-tetra­zoyl rings are quasi-coplanar, making a dihedral angle of 1.9 (2)°, while the uncoordinated rings show a larger inter­planar angle of 21.3 (2)°. The flexible propane spacer displays a zigzag chain. Inter­molecular O—H⋯N and O—H⋯O inter­actions result in two-dimensional polymeric structures parallel to (100). Two C atoms of the spacer are disordered over two positions, with site occupancy factors of *ca* 0.85 and 0.15.

## Related literature

For related literature, see: Fan *et al.* (2005 ▶); Gallardo *et al.* (2001 ▶, 2004 ▶); Gong *et al.* (2004 ▶); Mizukami *et al.* (2005 ▶); Rodríguez-Diéguez *et al.* (2007 ▶); Wang *et al.* (2005 ▶).
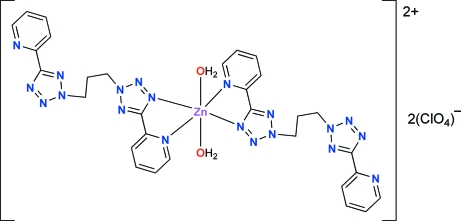


## Experimental

### 

#### Crystal data

[Zn(C_15_H_14_N_10_)(H_2_O)_2_](ClO_4_)_2_
*M* *_r_* = 969.03
Monoclinic, 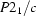

*a* = 7.378 (3) Å
*b* = 13.354 (3) Å
*c* = 20.764 (4) Å
β = 99.25 (2)°
*V* = 2019.2 (10) Å^3^
*Z* = 2
Mo *K*α radiation
μ = 0.82 mm^−1^
*T* = 293 (2) K
0.50 × 0.46 × 0.20 mm

#### Data collection

Enraf–Nonius CAD-4 diffractometer
Absorption correction: ψ scan (North *et al.*, 1968 ▶) *T*_min_ = 0.682, *T*_max_ = 0.853
3873 measured reflections
3576 independent reflections
2826 reflections with *I* > 2σ(*I*)
*R*_int_ = 0.016
3 standard reflections every 200 reflections intensity decay: 1%

#### Refinement

*R*[*F* ^2^ > 2σ(*F* ^2^)] = 0.035
*wR*(*F* ^2^) = 0.097
*S* = 1.06
3576 reflections
305 parameters
3 restraints
H-atom parameters constrained
Δρ_max_ = 0.30 e Å^−3^
Δρ_min_ = −0.41 e Å^−3^

### 

Data collection: *CAD-4 EXPRESS* (Enraf–Nonius, 1994 ▶); cell refinement: *SET4* in *CAD-4 EXPRESS*; data reduction: *HELENA* (Spek, 1996 ▶); program(s) used to solve structure: *SIR97* (Altomare *et al.*, 1999 ▶); program(s) used to refine structure: *SHELXL97* (Sheldrick, 2008 ▶); molecular graphics: *PLATON* (Spek, 2003 ▶) and *Mercury* (Macrae *et al.*, 2006 ▶); software used to prepare material for publication: *SHELXL97* and *publCIF* (Westrip, 2008 ▶).

## Supplementary Material

Crystal structure: contains datablocks global, I. DOI: 10.1107/S1600536808006703/bg2168sup1.cif

Structure factors: contains datablocks I. DOI: 10.1107/S1600536808006703/bg2168Isup2.hkl

Additional supplementary materials:  crystallographic information; 3D view; checkCIF report

## Figures and Tables

**Table 1:** Selected bond lengths (Å)

Zn1—O1*W*	2.1079 (18)
Zn1—N17	2.149 (2)
Zn1—N11	2.170 (2)

**Table 2:** Hydrogen-bond geometry (Å, °)

*D*—H⋯*A*	*D*—H	H⋯*A*	*D*⋯*A*	*D*—H⋯*A*
O1*W*—H1*WA*⋯O11	0.86	2.01	2.869 (3)	172
O1*W*—H1*WB*⋯N21^i^	0.86	1.97	2.833 (3)	178

Symmetry code: (i) 
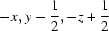
.

**Fig. 1.**
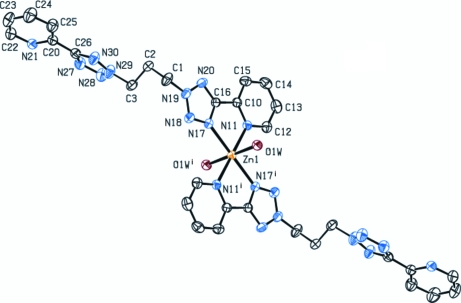
The molecular structure of the cation complex showing the labeling scheme. Displacement ellipsoids are shown at the 40% probability level. Symmetry code: (i) -x, -y, -z

**Fig. 2.**
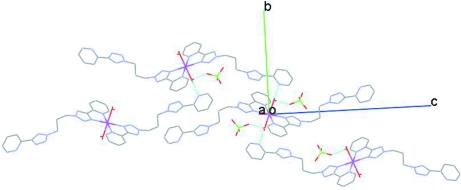
A detail of the two-dimensional polymeric strucuture formed by hydrogen bonding.

## supplementary crystallographic information

### Comment

New compounds for the research of supramolecular chemistry and crystal engineering have been extensively described in the literature in the last few years (Wang *et al.*, 2005; Fan *et al.*, 2005; Rodríguez-Diéguez *et al.*, 2007). Self-assembly processes involving organic ligands and metal ions have attracted much attention from the point of view of the development of novel functional materials with unique electronic, magnetic, catalytic and optical properties. However, while an accurate prediction of the overall crystal structure of such materials is not often an easy task, the introduction of rational organic ligands acting as building blocks has been recognized as a crucial synthetic strategy to overcome such difficulties. The syntheses of aromatic molecules containing nitrogen donor groups and which are interconnected by different type of spacers, such as conformationally rigid or flexible molecular skeletons, have been widely utilized as building blocks (Mizukami *et al.*, 2005; Gallardo *et al.*, 2001; Gong *et al.*, 2004).

The synthesis and X-ray crystal structure of the ligand 1,3-Bis-[(2-pyridyl)-2*H*-tetrazol-5-yl]propane (L1) has been described previously (Gallardo *et al.*, 2004). We report herein the title cation complex [Zn(L1)_2_(H_2_O)_2_]^2+^. The Zn^II^ atom lies on a center of symmetry and its distorted octahedral coordination is achieved through the interaction with four nitrogen atoms of two *trans* L1 ligands, defining the equatorial plane and two water molecules in apical positions (Fig. 1). The basal Zn1—N17 (2.149 (2) Å) and Zn1—N11 (2.170 (2) Å) distances are somewhat larger than the apical ones (Zn1—O1W: (2.108 (2) Å). Some conformational differences in the structure of the two 2-pyridyl-2*H*-tetrazoyl units in the L1 ligand can be observed: the coordination of the pyridyl and tetrazoyl rings of one of the units to the metal center imposes structural rigidity in this moiety, and the rings become rings quasi coplanar with an interplanar angle of 1.9 (2)°. The N11—C10—C16 angle (113.5 (2)°) is smaller than the expected value due to the restriction of the five-membered chelate ring. On the other hand, in the uncoordinated unit the bond the corresponding rings are free to rotate around C20—C26 and the interplanar angle between angle climbs up to 21.3 (2)°. Besides, the N21—C20—C26 angle (116.6 (2)°) is significantly larger than the one in the coordinated unit. A two-dimensional polymeric structure parallel to (100) is formed by intermolecular O—H···N interactions (Fig. 2). Finally, the perchlorate counterion is also connected to the polymeric structure by a O—H···O interaction.

### Experimental

Ligand L1 (obtained as described in Gallardo *et al.*, 2004) was added to a suspension of Zn(ClO)_4_.6H_2_O in Ethanol and stirred at 50°C for 30 min. The white product was filtered off and recrystallized from isopropyl alcohol/water (1:1) affording white crystals. Yield: 61%. Elemental analysis. Calc. C_30_H_32_Cl_2_N_20_O_10_Zn: C 37.18, H 3.33, N 28.91%. Found: C 37.27, H 3.29, N 28.98%.

### Refinement

H atoms attached to carbon atoms were added at their calculated positions and allowed to ride, with C—H_Ar_ = 0.93 Å and 0.97 Å for methylene groups and *U*_iso_(H) = 1.2*U*_eq_(C). H atoms of the water ligand were located from Fourier the difference map and treated in the riding model aproximation with *U*_iso_(H) fixed at 1.2 times of *U*_iso_(O). C2 and C3 atoms are disordered over two alternative positions which determine two different conformations for the propylene group. The occupancies for disordered atoms were refined and the respective values are 0.848 (8) and 0.152 (8).

### Crystal data

**Table:**

[Zn(C_15_H_14_N_10_)(H_2_O)_2_](ClO_4_)_2_	*F*_000_ = 992
*M**_r_* = 969.03	*D*_x_ = 1.594 Mg m^−^^3^
Monoclinic, *P*2_1_/*c*	Mo *K*α radiation λ = 0.71069 Å
Hall symbol: -P 2ybc	Cell parameters from 25 reflections
*a* = 7.378 (3) Å	θ = 9.6–15.4º
*b* = 13.354 (3) Å	µ = 0.82 mm^−^^1^
*c* = 20.764 (4) Å	*T* = 293 (2) K
β = 99.25 (2)º	Prismatic, colorless
*V* = 2019.2 (10) Å^3^	0.50 × 0.46 × 0.20 mm
*Z* = 2	

### Data collection

**Table:**

Enraf–Nonius CAD-4 diffractometer	*R*_int_ = 0.016
Radiation source: fine-focus sealed tube	θ_max_ = 25.1º
Monochromator: graphite	θ_min_ = 2.5º
*T* = 293(2) K	*h* = 0→8
ω–2θ scans	*k* = 0→15
Absorption correction: ψ scan(North *et al.*, 1968)	*l* = −24→24
*T*_min_ = 0.682, *T*_max_ = 0.853	3 standard reflections
3873 measured reflections	every 200 reflections
3576 independent reflections	intensity decay: 1%
2826 reflections with *I* > 2σ(*I*)	

### Refinement

**Table:**

Refinement on *F*^2^	Secondary atom site location: difference Fourier map
Least-squares matrix: full	Hydrogen site location: inferred from neighbouring sites
*R*[*F*^2^ > 2σ(*F*^2^)] = 0.035	H-atom parameters constrained
*wR*(*F*^2^) = 0.097	*w* = 1/[σ^2^(*F*_o_^2^) + (0.0463*P*)^2^ + 1.2496*P*] where *P* = (*F*_o_^2^ + 2*F*_c_^2^)/3
*S* = 1.06	(Δ/σ)_max_ < 0.001
3576 reflections	Δρ_max_ = 0.30 e Å^−^^3^
305 parameters	Δρ_min_ = −0.41 e Å^−^^3^
3 restraints	Extinction correction: none
Primary atom site location: structure-invariant direct methods	

### Fractional atomic coordinates and isotropic or equivalent isotropic displacement parameters (Å^2^)

**Table:**

	*x*	*y*	*z*	*U*_iso_*/*U*_eq_	Occ. (<1)
Zn1	0.0000	0.5000	0.5000	0.03313 (13)	
O1W	0.1357 (3)	0.37083 (13)	0.54150 (8)	0.0414 (4)	
H1WA	0.2079	0.3821	0.5779	0.050*	
H1WB	0.0630	0.3224	0.5479	0.050*	
C1	0.2499 (4)	0.4082 (2)	0.26479 (13)	0.0525 (8)	
H1C	0.3610	0.4474	0.2763	0.063*	0.848 (8)
H1B	0.2857	0.3387	0.2618	0.063*	0.848 (8)
H1A'	0.3789	0.4082	0.2838	0.063*	0.152 (8)
H1B'	0.2230	0.3455	0.2416	0.063*	0.152 (8)
C2	0.1502 (6)	0.4409 (3)	0.19841 (17)	0.0478 (12)	0.848 (8)
H2A	0.0335	0.4063	0.1884	0.057*	0.848 (8)
H2B	0.2233	0.4235	0.1652	0.057*	0.848 (8)
C2'	0.212 (2)	0.5004 (19)	0.2178 (9)	0.062 (9)	0.152 (8)
H2A'	0.3042	0.4990	0.1893	0.075*	0.152 (8)
H2B'	0.2302	0.5620	0.2428	0.075*	0.152 (8)
C3	0.1192 (7)	0.5522 (3)	0.19766 (16)	0.0473 (11)	0.848 (8)
H3A	0.0221	0.5680	0.2224	0.057*	0.848 (8)
H3B	0.2299	0.5865	0.2177	0.057*	0.848 (8)
C3'	0.031 (2)	0.5010 (16)	0.1744 (9)	0.048 (6)	0.152 (8)
H3A'	−0.0017	0.4382	0.1521	0.058*	0.152 (8)
H3B'	−0.0640	0.5198	0.1994	0.058*	0.152 (8)
C10	−0.2250 (3)	0.36570 (18)	0.40158 (11)	0.0336 (5)	
N11	−0.2337 (3)	0.40794 (15)	0.46025 (9)	0.0330 (5)	
C12	−0.3779 (4)	0.3837 (2)	0.48910 (13)	0.0407 (6)	
H12	−0.3880	0.4129	0.5290	0.049*	
C13	−0.5122 (4)	0.3170 (2)	0.46167 (15)	0.0488 (7)	
H13	−0.6099	0.3013	0.4831	0.059*	
C14	−0.4989 (4)	0.2741 (2)	0.40210 (15)	0.0505 (7)	
H14	−0.5869	0.2285	0.3831	0.061*	
C15	−0.3538 (4)	0.2996 (2)	0.37130 (13)	0.0433 (6)	
H15	−0.3432	0.2727	0.3308	0.052*	
C16	−0.0630 (3)	0.39652 (18)	0.37349 (11)	0.0333 (5)	
N17	0.0622 (3)	0.45884 (16)	0.40591 (10)	0.0351 (5)	
N18	0.1900 (3)	0.47270 (17)	0.36914 (10)	0.0404 (5)	
N19	0.1370 (3)	0.41780 (17)	0.31670 (10)	0.0395 (5)	
N20	−0.0198 (3)	0.36842 (17)	0.31667 (10)	0.0414 (5)	
C20	0.1645 (4)	0.6352 (2)	−0.02723 (13)	0.0393 (6)	
N21	0.1096 (3)	0.71610 (16)	−0.06368 (11)	0.0429 (5)	
C22	0.1892 (4)	0.7317 (2)	−0.11638 (14)	0.0534 (8)	
H22	0.1536	0.7876	−0.1420	0.064*	
C23	0.3205 (5)	0.6698 (3)	−0.13475 (16)	0.0654 (9)	
H23	0.3741	0.6845	−0.1712	0.078*	
C24	0.3713 (5)	0.5852 (3)	−0.09787 (17)	0.0697 (10)	
H24	0.4573	0.5409	−0.1098	0.084*	
C25	0.2923 (4)	0.5676 (2)	−0.04327 (15)	0.0552 (8)	
H25	0.3242	0.5113	−0.0176	0.066*	
C26	0.0884 (4)	0.62351 (19)	0.03362 (13)	0.0394 (6)	
N27	−0.0628 (3)	0.66990 (18)	0.04770 (12)	0.0485 (6)	
N28	−0.0761 (4)	0.64443 (19)	0.10824 (12)	0.0540 (6)	
N29	0.0649 (4)	0.58592 (19)	0.12805 (12)	0.0539 (6)	
N30	0.1722 (3)	0.56958 (19)	0.08358 (11)	0.0513 (6)	
Cl1	0.34362 (10)	0.38846 (6)	0.71439 (3)	0.0497 (2)	
O11	0.3975 (4)	0.4197 (2)	0.65481 (12)	0.0874 (8)	
O12	0.4636 (4)	0.3152 (2)	0.74296 (16)	0.1075 (11)	
O13	0.1623 (4)	0.3525 (3)	0.70176 (15)	0.1099 (11)	
O14	0.3557 (5)	0.4722 (3)	0.75703 (19)	0.1274 (13)	

### Atomic displacement parameters (Å^2^)

**Table:**

	*U*^11^	*U*^22^	*U*^33^	*U*^12^	*U*^13^	*U*^23^
Zn1	0.0396 (2)	0.0337 (2)	0.0263 (2)	−0.00564 (18)	0.00595 (16)	−0.00305 (17)
O1W	0.0500 (11)	0.0362 (10)	0.0370 (10)	−0.0037 (8)	0.0033 (8)	0.0006 (8)
C1	0.0586 (18)	0.066 (2)	0.0379 (15)	0.0157 (16)	0.0229 (14)	0.0050 (14)
C2	0.067 (3)	0.048 (2)	0.0299 (19)	−0.006 (2)	0.0123 (19)	−0.0028 (16)
C2'	0.028 (10)	0.13 (3)	0.028 (10)	0.024 (15)	0.012 (8)	0.024 (15)
C3	0.061 (3)	0.050 (2)	0.0314 (19)	0.004 (2)	0.0107 (19)	−0.0017 (16)
C3'	0.038 (11)	0.067 (15)	0.042 (11)	0.008 (11)	0.009 (9)	0.025 (11)
C10	0.0382 (14)	0.0300 (13)	0.0308 (12)	0.0017 (11)	0.0003 (10)	0.0012 (10)
N11	0.0355 (11)	0.0312 (11)	0.0319 (11)	−0.0005 (9)	0.0043 (9)	0.0027 (9)
C12	0.0395 (14)	0.0410 (15)	0.0422 (14)	0.0031 (12)	0.0087 (12)	0.0041 (12)
C13	0.0338 (14)	0.0504 (17)	0.0623 (19)	−0.0011 (13)	0.0082 (13)	0.0105 (15)
C14	0.0404 (15)	0.0460 (17)	0.0608 (19)	−0.0081 (13)	−0.0045 (14)	−0.0024 (14)
C15	0.0445 (15)	0.0411 (15)	0.0420 (15)	−0.0022 (12)	0.0000 (12)	−0.0062 (12)
C16	0.0401 (14)	0.0293 (12)	0.0294 (12)	0.0008 (11)	0.0026 (11)	0.0015 (10)
N17	0.0405 (12)	0.0364 (11)	0.0295 (11)	−0.0032 (10)	0.0088 (9)	0.0005 (9)
N18	0.0467 (13)	0.0425 (13)	0.0332 (12)	0.0003 (10)	0.0102 (10)	0.0000 (9)
N19	0.0478 (13)	0.0429 (12)	0.0291 (11)	0.0050 (11)	0.0099 (10)	0.0025 (9)
N20	0.0505 (14)	0.0447 (13)	0.0287 (11)	0.0032 (11)	0.0060 (10)	−0.0049 (10)
C20	0.0429 (15)	0.0370 (14)	0.0373 (14)	−0.0021 (12)	0.0045 (12)	0.0000 (11)
N21	0.0537 (14)	0.0359 (12)	0.0386 (12)	−0.0020 (11)	0.0054 (11)	0.0000 (10)
C22	0.067 (2)	0.0500 (18)	0.0426 (16)	−0.0033 (15)	0.0075 (15)	0.0075 (14)
C23	0.069 (2)	0.086 (3)	0.0469 (17)	−0.0004 (19)	0.0247 (16)	0.0100 (18)
C24	0.068 (2)	0.081 (2)	0.066 (2)	0.0221 (19)	0.0282 (18)	0.0054 (19)
C25	0.0602 (19)	0.0541 (19)	0.0534 (18)	0.0129 (16)	0.0153 (15)	0.0094 (15)
C26	0.0448 (15)	0.0314 (14)	0.0418 (15)	0.0009 (12)	0.0066 (12)	−0.0003 (11)
N27	0.0533 (15)	0.0432 (13)	0.0511 (14)	0.0121 (11)	0.0145 (12)	0.0072 (11)
N28	0.0587 (16)	0.0535 (15)	0.0535 (15)	0.0171 (13)	0.0207 (13)	0.0091 (12)
N29	0.0618 (16)	0.0559 (15)	0.0484 (14)	0.0193 (13)	0.0228 (12)	0.0112 (12)
N30	0.0565 (15)	0.0568 (15)	0.0440 (13)	0.0168 (12)	0.0189 (12)	0.0100 (12)
Cl1	0.0474 (4)	0.0528 (4)	0.0465 (4)	0.0002 (3)	0.0006 (3)	0.0051 (3)
O11	0.0928 (19)	0.109 (2)	0.0586 (15)	−0.0223 (17)	0.0069 (14)	0.0235 (15)
O12	0.104 (2)	0.095 (2)	0.113 (2)	0.0239 (18)	−0.0123 (19)	0.0464 (19)
O13	0.0612 (17)	0.168 (3)	0.098 (2)	−0.0375 (19)	0.0047 (15)	−0.009 (2)
O14	0.141 (3)	0.111 (2)	0.137 (3)	−0.016 (2)	0.042 (3)	−0.068 (2)

### Geometric parameters (Å, °)

**Table:**

Zn1—O1W^i^	2.1079 (18)	C12—H12	0.9300
Zn1—O1W	2.1079 (18)	C13—C14	1.381 (4)
Zn1—N17^i^	2.149 (2)	C13—H13	0.9300
Zn1—N17	2.149 (2)	C14—C15	1.375 (4)
Zn1—N11^i^	2.170 (2)	C14—H14	0.9300
Zn1—N11	2.170 (2)	C15—H15	0.9300
O1W—H1WA	0.8646	C16—N20	1.325 (3)
O1W—H1WB	0.8638	C16—N17	1.341 (3)
C1—N19	1.470 (3)	N17—N18	1.319 (3)
C1—C2	1.519 (4)	N18—N19	1.319 (3)
C1—C2'	1.569 (17)	N19—N20	1.331 (3)
C1—H1C	0.9700	C20—N21	1.343 (3)
C1—H1B	0.9700	C20—C25	1.385 (4)
C1—H1A'	0.9700	C20—C26	1.471 (4)
C1—H1B'	0.9700	N21—C22	1.339 (4)
C2—C3	1.503 (5)	C22—C23	1.373 (5)
C2—H2A	0.9700	C22—H22	0.9300
C2—H2B	0.9699	C23—C24	1.383 (5)
C2'—C3'	1.485 (17)	C23—H23	0.9300
C2'—H2A'	0.9700	C24—C25	1.375 (4)
C2'—H2B'	0.9700	C24—H24	0.9300
C3—N29	1.505 (4)	C25—H25	0.9300
C3—H3A	0.9701	C26—N30	1.331 (3)
C3—H3B	0.9700	C26—N27	1.348 (3)
C3'—N29	1.534 (14)	N27—N28	1.321 (3)
C3'—H3A'	0.9699	N28—N29	1.313 (3)
C3'—H3B'	0.9700	N29—N30	1.327 (3)
C10—N11	1.353 (3)	Cl1—O12	1.387 (3)
C10—C15	1.373 (4)	Cl1—O13	1.406 (3)
C10—C16	1.470 (3)	Cl1—O14	1.420 (3)
N11—C12	1.342 (3)	Cl1—O11	1.421 (3)
C12—C13	1.385 (4)		
			
O1W^i^—Zn1—O1W	180.00 (9)	C10—N11—Zn1	115.41 (16)
O1W^i^—Zn1—N17^i^	90.32 (8)	N11—C12—C13	122.5 (3)
O1W—Zn1—N17^i^	89.68 (8)	N11—C12—H12	118.7
O1W^i^—Zn1—N17	89.68 (8)	C13—C12—H12	118.7
O1W—Zn1—N17	90.32 (8)	C14—C13—C12	119.1 (3)
N17^i^—Zn1—N17	180.00 (4)	C14—C13—H13	120.5
O1W^i^—Zn1—N11^i^	89.32 (8)	C12—C13—H13	120.5
O1W—Zn1—N11^i^	90.68 (8)	C15—C14—C13	119.1 (3)
N17^i^—Zn1—N11^i^	77.40 (8)	C15—C14—H14	120.5
N17—Zn1—N11^i^	102.60 (8)	C13—C14—H14	120.5
O1W^i^—Zn1—N11	90.68 (8)	C10—C15—C14	118.7 (3)
O1W—Zn1—N11	89.32 (8)	C10—C15—H15	120.7
N17^i^—Zn1—N11	102.60 (8)	C14—C15—H15	120.7
N17—Zn1—N11	77.40 (8)	N20—C16—N17	112.2 (2)
N11^i^—Zn1—N11	180.00 (9)	N20—C16—C10	127.0 (2)
Zn1—O1W—H1WA	113.6	N17—C16—C10	120.9 (2)
Zn1—O1W—H1WB	114.1	N18—N17—C16	107.2 (2)
H1WA—O1W—H1WB	107.9	N18—N17—Zn1	140.15 (17)
N19—C1—C2	113.0 (3)	C16—N17—Zn1	112.61 (16)
N19—C1—C2'	108.8 (8)	N17—N18—N19	104.8 (2)
N19—C1—H1C	109.1	N18—N19—N20	114.7 (2)
C2—C1—H1C	109.6	N18—N19—C1	121.8 (2)
N19—C1—H1B	108.9	N20—N19—C1	123.4 (2)
C2—C1—H1B	108.3	C16—N20—N19	101.1 (2)
H1C—C1—H1B	107.8	N21—C20—C25	123.0 (3)
N19—C1—H1A'	109.6	N21—C20—C26	116.6 (2)
C2'—C1—H1A'	108.6	C25—C20—C26	120.4 (2)
C2'—C1—H1B'	111.6	C22—N21—C20	116.9 (2)
H1A'—C1—H1B'	108.1	N21—C22—C23	123.8 (3)
C3—C2—C1	110.2 (3)	N21—C22—H22	118.1
C3—C2—H2A	109.9	C23—C22—H22	118.1
C1—C2—H2A	110.0	C22—C23—C24	118.6 (3)
C3—C2—H2B	109.3	C22—C23—H23	120.7
C1—C2—H2B	109.4	C24—C23—H23	120.7
H2A—C2—H2B	108.0	C25—C24—C23	118.9 (3)
C3'—C2'—C1	115.7 (15)	C25—C24—H24	120.6
C3'—C2'—H2A'	106.3	C23—C24—H24	120.6
C1—C2'—H2A'	106.9	C24—C25—C20	118.8 (3)
C3'—C2'—H2B'	110.7	C24—C25—H25	120.6
C1—C2'—H2B'	109.7	C20—C25—H25	120.6
H2A'—C2'—H2B'	107.1	N30—C26—N27	112.1 (2)
C2—C3—N29	108.8 (3)	N30—C26—C20	122.2 (2)
C2—C3—H3A	109.5	N27—C26—C20	125.5 (2)
N29—C3—H3A	109.8	N28—N27—C26	106.2 (2)
C2—C3—H3B	110.3	N29—N28—N27	106.0 (2)
N29—C3—H3B	110.2	N28—N29—N30	114.3 (2)
H3A—C3—H3B	108.2	N28—N29—C3	123.6 (3)
C2'—C3'—N29	99.4 (12)	N30—N29—C3	121.5 (3)
C2'—C3'—H3A'	114.6	N28—N29—C3'	115.9 (7)
N29—C3'—H3A'	113.1	N30—N29—C3'	119.4 (9)
C2'—C3'—H3B'	109.5	N29—N30—C26	101.5 (2)
N29—C3'—H3B'	110.4	O12—Cl1—O13	111.2 (2)
H3A'—C3'—H3B'	109.4	O12—Cl1—O14	108.5 (2)
N11—C10—C15	123.4 (2)	O13—Cl1—O14	110.3 (2)
N11—C10—C16	113.5 (2)	O12—Cl1—O11	109.3 (2)
C15—C10—C16	123.0 (2)	O13—Cl1—O11	109.19 (18)
C12—N11—C10	117.2 (2)	O14—Cl1—O11	108.3 (2)
C12—N11—Zn1	127.24 (17)		

Symmetry codes: (i) −*x*, −*y*+1, −*z*+1.

### Hydrogen-bond geometry (Å, °)

**Table:**

*D*—H···*A*	*D*—H	H···*A*	*D*···*A*	*D*—H···*A*
O1W—H1WA···O11	0.86	2.01	2.869 (3)	172
O1W—H1WB···N21^ii^	0.86	1.97	2.833 (3)	178

Symmetry codes: (ii) −*x*, *y*−1/2, −*z*+1/2.
